# Account of Diseases in an Eastern District of London, from the 20th of June, to the 20th of July

**Published:** 1799-08

**Authors:** 


### STATE OF DISEASES IN LONDON.

*Account of Diſeaſes in an Eaſtern Diſtrict of London, from the 20th of June, to the 20th of July.*

| ACUTE DISEASES. | No. of Cases. |
|---|---|
| Typhus | 3 |
| Quotidian | 1 |
| Meaſles | 3 |
| Scarlatina | 2 |
| Acute Rheumatiſm | 3 |
| **CHRONIC DISEASES.** | |
| Cough | 4 |
| Dyſpnœa | 5 |
| Cough and Dyſpnœa | 6 |
| Aſthma | 2 |
| Phthiſis Pulmonalis | 5 |
| Pleurodyne | 2 |
| Hæmoptoe | 1 |
| Hydrothorax | 3 |
| Aſcites | 5 |
| Cephalalgia | 3 |
| Apoplexy | 2 |
| Hemiplegia | 3 |
| Epilepſy | 1 |
| Vertigo | 4 |
| Epiſtaxis | 3 |
| Dyſpepſia | 6 |
| Vomitus | 2 |
| Gaſtrodynia | 8 |
| Enterodynia | 6 |

| | No. of Cases. |
|---|---|
| Amenorrhœa | 4 |
| Menorrhagia difficilis | 2 |
| Chloroſis | 3 |
| Hæmorrhois | 2 |
| Calculus | 1 |
| Dyſuria | 6 |
| Fluor albus | 7 |
| Scrophula | 5 |
| Hyſteria | 3 |
| Palpitatio | 2 |
| Hypochondriaſis | 3 |
| Chronic Rheumatiſm | 11 |
| Gout | 1 |
| **PUERPERAL DISEASES.** | |
| Dolores poſt partum | 3 |
| Enureſis | 1 |
| Maſtrodynia | 8 |
| Abſceſſus Mammarum | 2 |
| **INFANTILE DISEASES.** | |
| Ophthalmia | 3 |
| Ophthalmia purulenta | 2 |
| Aphthæ | 9 |
| Convulſio | 2 |
| Tooth raſh | 2 |
| Rachitis | 2 |

The meaſles which have lately occurred, have proved a ſlight diſeaſe, ſo that in ſome inſtances the patient has hardly required any medical aſſiſtance. The fever has been very inconſiderable and the different catarrhal ſymptoms have been juſt ſufficient to characterize the diſeaſe. The eruption has made its appearance at the time and has gradually diſappeared, in ſome caſes, without leaving any conſiderable degree of pneumonic affection. This termination does not always take place in the diſeaſe, when it is in other reſpects favourable; ſo that a caution is neceſſary againſt too ſoon taking it for granted, that all conſequences of the diſeaſe are over, when it has gone through its regular ſtages. It has ſometimes been obſerved that, where the diſeaſe has been of the milder kind, the ſucceeding ſymptoms of the inflammatory and pneumonic affection have been very ſevere, and have produced conſequences that have ultimately proved fatal.

In the treatment of this diſeaſe the antiphlogiſtic plan muſt be obſerved. In ſome caſes the free uſe of the lancet has been neceſſary; though in others this neceſſity has been ſuperſeded by adminiſtering the cooling purgatives and antimonial remedies, and obſerving a ſtrictly antiphlogiſtic regimen. The cough may be palliated by demulcent remedies, to which, if there be not much fever, opiates may be added. Where the uſe of the lancet has been diſpenſed with, the application of leeches to the cheſt has ſometimes been found an expedient practice, and a bliſter applied to the ſternum has relieved under the prevalence of cough and difficult reſpiration.

Aphthæ in children have lately been more than uſually prevalent, and in ſome caſes, have proved very obſtinate. This diſorder is very common, and very well known by thoſe who have the care of infants. It appears on the lips, the tongue, and different parts of the fauces, in little white ſpecks; which in ſome caſes unite ſo cloſely as to form a kind of cruſt, covering the whole inſide of the mouth and throat.

The firſt crop is ſometimes ſucceeded by a ſecond: this, though it may ſometimes take place in the natural courſe of the diſeaſe, is often occaſioned by an early and injudicious attempt to remove the cruſt by ſome topical applications. To keep the bowels open by gentle laxatives, and to correct the acidity which frequently prevails, by the teſtaceous powders, is perhaps the moſt proper plan of treatment.

